# Dissecting Heterogeneity Reveals Functional Subpopulation of Stem Cells From Human Exfoliated Deciduous Teeth

**DOI:** 10.1016/j.identj.2025.104014

**Published:** 2025-11-06

**Authors:** Yuqing Peng, Yanqiang Zhao, Dan Wang, Jingyi Cui, Yijia Qiu, Man Qin, Yuanyuan Wang

**Affiliations:** Department of Paediatric Dentistry, School and Hospital of Stomatology, Peking University, Beijing, China

**Keywords:** BAMBI, Heterogeneity, Single-cell RNA sequencing, Stem cells from human exfoliated deciduous teeth

## Abstract

**Objectives:**

Identify the functional subpopulation’s surface marker of stem cells from human exfoliated deciduous teeth (SHEDs) using single-cell RNA sequencing (scRNA-seq) and isolate the subset, providing a theoretical basis for the refined application of stem cells.

**Methods:**

scRNA-seq identified SHEDs as distinct subpopulations. GO and KEGG analyses were performed to define each subpopulation. Pseudotime trajectory analysis was employed to elucidate the potential differentiation processes of SHEDs. To investigate the multipotent differentiation potential of the BAMBI^+^ subpopulation, we conducted qPCR, Western blot analysis, Alizarin Red staining, Oil Red O staining, Alcian Blue staining, and immunohistochemical staining. The angiogenic capacity of the BAMBI^+^ subpopulation was also verified through cratch tests, transwell migration assays, qPCR, Matrigel tube formation assays, and chick embryo chorioallantoic membrane (CAM) assay.

**Results:**

Major subpopulations of SHEDs, including a proliferative subpopulation, a functional subpopulation, fibroblast-like cells, were identified. As for the transformation trajectories, the proliferative subpopulation is the root population, which then differentiates into a functional subpopulation or fibroblast-like cells. The functional subpopulation is characterized by BAMBI expression and exhibits strong angiogenic, chondrogenic, and proliferative capabilities. Cells at different passages presented distinct and common characteristics.

**Conclusions:**

SHEDs constitute a heterogeneous population and BAMBI⁺ subpopulation has higher proliferative, chondrogenic and angiogenic capacities.

**Clinical relevance:**

This study offers a theoretical basis for optimizing SHED-based cell therapeutic strategies. BAMBI⁺ SHEDs, excelling in angiogenesis/chondrogenesis, may be used for tissue-engineered therapies of the clinical disorders such as temporomandibular joint disc regeneration via “vascularize-then-cartilage” strategy.

## Introduction

Mesenchymal stem cells (MSCs) are a type of adult stem cell that possesses the ability for self-renewal, multipotent differentiation, and immune regulation. MSCs have attracted substantial attention owing to their potential in tissue regeneration and disease treatment. However, the heterogeneity of MSCs and their functional differences across tissues limit their effectiveness in clinical applications. Stem cells from human exfoliated deciduous teeth (SHEDs) are a type of MSC with multipotent differentiation potential. ^1^ They can be easily obtained from naturally shed deciduous teeth, offering advantages such as abundant sources, convenient acquisition, and minimal ethical concerns.^2^^,^^3^ SHEDs not only have the capacity to differentiate into odontoblasts and osteoblasts but also exhibit strong abilities to promote angiogenesis and neurogenesis, making them highly promising for applications in regenerative medicine.^4^^,^^5^ However, research on the heterogeneity and functional subgroups of SHEDs remains relatively limited.

The advent of scRNA-seq technology has provided a powerful tool for studying cellular heterogeneity. scRNA-seq enables comprehensive analysis of the transcriptomes of cells at the single-cell level, revealing the heterogeneity and dynamic changes of cell subpopulations. ^6^ In recent years, scRNA-seq has been widely applied to the study of various types of MSCs, including bone marrow-derived MSCs (BMSCs), umbilical cord-derived MSCs (UCMSCs), and adipose tissue-derived MSCs (ADSCs).^7^, ^8^, ^9^ These studies have revealed the heterogeneity of MSCs from different tissue sources at the transcriptomic level and have provided important insights into their functional differences.

Recent studies have used scRNA-seq technology to provide a comprehensive overview of the heterogeneity of human dental pulp-derived MSCs.^10^, ^11^ Crucially, SHEDs (stem cells from human exfoliated deciduous teeth) and DPSCs (dental pulp stem cells) are fundamentally distinct cell populations with unique transcriptomic profiles and differentiation potentials. ^1^ However, despite the great potential of SHEDs in regenerative medicine, systematic studies on their heterogeneity and functional subgroups are still in their infancy. Our study aims to comprehensively analyze the transcriptional heterogeneity of SHEDs using scRNA-seq technology. For the first time, we have generated a comprehensive single-cell map of SHEDs across different passages and studied the transformation trajectories of each subgroup. We found that the BAMBI^+^ subgroup has unique functional characteristics. This study not only provides new insights into the heterogeneity and functional properties of SHEDs but also offers a theoretical basis for optimizing SHED-based cell therapy strategies.

## Materials and methods

### Isolation and culture of SHEDs

All experimental protocols were approved by the Ethics Committee of the Peking University Health Science Center, Beijing, China (ethical approval number: PKUSSIRB-202394170). SHEDs were harvested from primary teeth that were extracted at the Department of Paediatric Dentistry, Peking University School and Hospital of Stomatology. The procedure involved removal of the dental crown at the cervical level to access the pulp tissue. The pulp was then minced and enzymatically dissociated using a solution containing 3 mg/mL type I collagenase (Sigma‒Aldrich) and 4 mg/mL dispase (Sigma‒Aldrich) for 1 hour at 37 °C. The SHED suspensions were subsequently filtered through a 70-mm mesh strainer (Falcon) to isolate individual cells. The resulting single-cell suspensions were cultured in a-MEM (GIBCO/BRL) supplemented with 10% foetal bovine serum (GIBCO), 100 U/mL penicillin, and 100 mg/mL streptomycin. The cultures were maintained at 37 °C with 5% CO_2_, and the medium was changed every 3 days. When the cultures reached 90% confluence, the SHEDs were either passaged using 0.25% trypsin containing 0.53 mM EDTA for single-cell experiments or reseeded into new flasks for further expansion.

### Single-cell RNA sequencing

Three SHED samples at passage 0, passage 5, and passage 10 were prepared for scRNA-seq assays. The SHEDs were digested into a single-cell suspension. The cell suspension was prepared according to the manufacturer’s protocols. The sample was loaded into a 10X Chromium controller, and library preparation was performed. The resulting libraries were sequenced in an Illumina NovaSeq sequencer according to 10X Genomics recommendations to a depth of approximately 100,000 reads per cell. We have uploaded our original sequencing data to the GSA-human database (no. HRA011999).

### Quality control and analysis

Expression matrix files for subsequent analyses were generated from gene counts and unique molecular identifier (UMI) counts. Any batch effects between our sequencing sample data and data from the public database were removed by Harmony. The cells were screened according to a gene count in the range of 300 and a UMI count under 30,000. Cells containing more than 10% mitochondrial content were excluded.

Further analysis, including data normalization, cell cycle correction, dimensionality reduction, and K-means cell clustering, was performed using R (version 4.1.2) with the Seurat package (version 4.4.0). The SingleR package annotates cell types by calculating correlation coefficients between the expression profile of query cells and a reference single-cell RNA sequencing dataset, then assigning the cell type from the reference dataset that shows the highest correlation to each query cell. Gene Ontology (GO) and Kyoto Encyclopedia of Genes and Genomes (KEGG) analyses were performed using DAVID Bioinformatics Resources (version 6.8). Monocle was employed to construct single-cell trajectory analysis, incorporating pseudotime. The degree of differentiation of each cell subset was calculated using the R package CytoTRACE (version 1.8.0). Genes were filtered with the following criteria: expressed in more than 10 cells, an average expression value greater than 0.1, and a Qval less than 0.01 in the analyses. All data analysis was performed in cooperation with Singleron Biotechnologies (Nanjing, China).

### Immunofluorescence staining and immunohistochemistry

Cells were fixed in 4% paraformaldehyde, permeabilized with 0.4% Triton X-100, blocked with 3% BSA, and incubated with anti-BAMBI antibody (1:200, Bioss, bs-12418R-Cy5) overnight at 4 °C. They were labeled with Alexa Fluor 488-conjugated goat anti-mouse IgG (1:1000, CST) and counterstained with DAPI (1:500). Images were acquired on a Nikon microscope.

For immunohistochemistry, paraffin-embedded tissue sections were deparaffinized, rehydrated, and stained with anti-COL1 antibody (1:200, CST). The sections were then visualized using a standard immunohistochemistry protocol and imaged with a light microscope.

### Flow cytometry analysis and cell sorting

To prepare SHEDs for cell sorting, the cells were first rinsed with PBS and then digested with 0.5 mM EDTA for 3 minutes. The digestion was subsequently terminated, and the cell suspension was thoroughly pipetted to ensure complete dissociation before filtration through a 70-μm cell strainer to collect single cells. The cells were then centrifuged and resuspended (10^5^ for analysis and 10^7^ for sorting) in cell culture medium containing appropriate antibodies. An anti-human BAMBI antibody conjugated with Cy5 (1:200, bs12418R-Cy5, Bioss) was added to the cell suspension, followed by incubation for 30 minutes in the dark. After incubation, the cells were centrifuged to collect the cell pellets. The supernatant was carefully removed, and the cells were washed three times with PBS before being detected or subjected to cell sorting using an Aria III FACS machine (Becton Dickinson). A PBS tube was used as a blank control, and single-stained tubes were used as compensation controls. The data were analyzed using FlowJo^TM^ software (Ashland, OR).

### Cell proliferation assay

The cells were seeded in 96-well plates at a density of 3000 cells per well. On days 1, 3, 5, and 7 after cell seeding, cell viability was evaluated using a CCK-8 assay (Dojindo, Japan) according to the manufacturer’s instructions. The absorbance at 450 nm was measured, and proliferation curves were plotted. At least three replicate wells per concentration were examined.

We also performed colony formation experiments to confirm the ability of the cells to proliferate. In these experiments, 300 cells were seeded in each well of 12-well plates and cultured for 10 days, and the colonies were counted after staining with 0.2% crystal violet for 10 min. This experiment was repeated three times.

### Multilineage differentiation of SHEDs

For osteogenic differentiation, the cells were cultured in basal 10% FBS/α-MEM supplemented with 0.01 mM dexamethasone disodium phosphate, 0.1 mM L-ascorbic acid phosphate, and 1.8 mM monobasic potassium phosphate (Sigma‒Aldrich, USA) as we previously did^12^. Mineralization was detected by staining with 2% w/v Alizarin Red S on day 21. Quantification was then performed using ImageJ software.

To induce adipogenic and chondrogenic differentiation, the cells were cultured with a Human Stem Cell Adipogenic Differentiation Kit (Oricell, China) and a Human Stem Cell Chondrogenic Differentiation Kit (Oricell, China) according to the manufacturer’s protocols. Two to four weeks later, the cells were fixed for Oil Red O staining. Isopropanol was added, and the mixture was gently agitated to dissolve the sample, which was then transferred to a 96-well plate. The absorbance at 590 nm was measured using a microplate reader. The “cartilage pellet” was fixed and paraffin-embedded, and Alcian blue staining was performed. For quantification, the pellets from three additional cultures were fixed and stained. The Alcian blue dye was subsequently extracted overnight using 4 mol·L^–1^ guanidine HCl (pH 5.8), and its absorbance was measured at 590 nm. Other sections were immunostained with monoclonal antibodies against human COL1 (1:200, Abcam, UK).

### Angiogenic potential of SHEDs

The angiogenic induction medium was α-MEM supplemented with 50 μg/L VEGF, 10 μg/L b-FGF, and 10% FBS. After 2 weeks of induction, the cells were seeded into 24-well plates precoated with Matrigel (BD, USA). Images were captured after 6 hours of culture.

To explore the effects of BAMBI^+^ SHEDs on the migration capacity of human umbilical vein endothelial cells (HUVECs), scratch tests and transwell migration assays were performed. BAMBI^+^ SHEDs and BAMBI^-^ SHEDs were separately cultured in complete medium for 7 days, followed by centrifugation to collect the supernatant, which was then used as cell culture media for subsequent experiments. Then confluent monolayers of HUVECs with different cell culture media were subjected to a scratch along the diameter of the well using a pipette tip. The movement of the cells into the denuded area was monitored after 24 hours. As for the transwell migration assays, after the upper chamber of the Transwell chamber (Corning, USA) was moistened with PBS, HUVECs were seeded into the upper chamber, and the experimental medium (BAMBI^+^ SHEDs and BAMBI^-^ SHEDs culture media) was added to the lower chamber. Following a 24-h incubation period, the cells were fixed and stained with 0.1% crystal violet for observation. Quantitative analysis was carried out using ImageJ software.

The fertilized eggs were incubated at 38.3 °C for the first three days, followed by incubation at 38.5 °C for the next two days in a humid environment. After 7 days, a small hole was drilled in the eggshell to expose the chorioallantoic membrane (CAM) to the cell culture medium (BAMBI^+^ SHEDs and BAMBI^-^ SHEDs culture media; the preparation method was the same as described above). Before the eggs were returned to the incubator, the opening was covered with sterile paraffin film. Five days later, the CAM was carefully removed from the eggs to assess angiogenesis. Images were captured using a stereomicroscope to quantify angiogenesis, and new blood vessel branches were independently counted twice in a double-blinded manner.

### Quantitative real-time polymerase chain reaction (qRT‒PCR)

Total RNA from SHEDs was isolated using TRIzol reagent (Invitrogen, USA), followed by cDNA synthesis using the PrimeScript RT Reagent Kit (TaKaRa, Shiga, Japan). The qPCR assay for mRNA detection was performed with a SYBR Green Kit (Roche). GAPDH was used as an internal reference. The relative expression levels of each gene were analysed using the 2^−△△^C_t_ method. The primers used for qRT‒PCR are shown in the Table S1.

### Western blot analysis

The cells were incubated in induction medium for 1–2 weeks and then lysed in RIPA buffer containing protease and phosphatase inhibitors. Protein was extracted and quantified using a BCA protein assay (Pierce). Briefly, protein from each sample was separated and then transferred to PVDF membranes (Millipore). The membranes were incubated in blocking buffer (5% nonfat dry milk) and then incubated with the following antibodies: anti-SOX9 (1:1000, Abcam), and anti-PPARG (1:1000, Abcam). The membranes were then incubated with a horseradish peroxidase-conjugated secondary antibody (PV9001, PV9002, ZSJQ). The protein bands were visualized using a Fusin Fx assay (Vilber Lourmat).

### Statistical analysis

Statistical calculations were performed using GraphPad Prism 8. Comparisons between two groups were performed using an independent two-tailed Student’s t test, and comparisons among > 2 groups were analysed using one-way analysis of variance followed by Tukey’s post hoc test. All the data are expressed as the means ± SDs of three experiments per group, and *P* < 0.05 was considered to indicate statistical significance (^∗^*P* < 0.05, ^∗∗^*P* < 0.01, ^∗∗∗^*P* < 0.001, ^∗∗∗∗^*P* < 0.0001).

## Results

### scRNA-seq of SHEDs identified distinct subpopulations

**Fig. 1:**
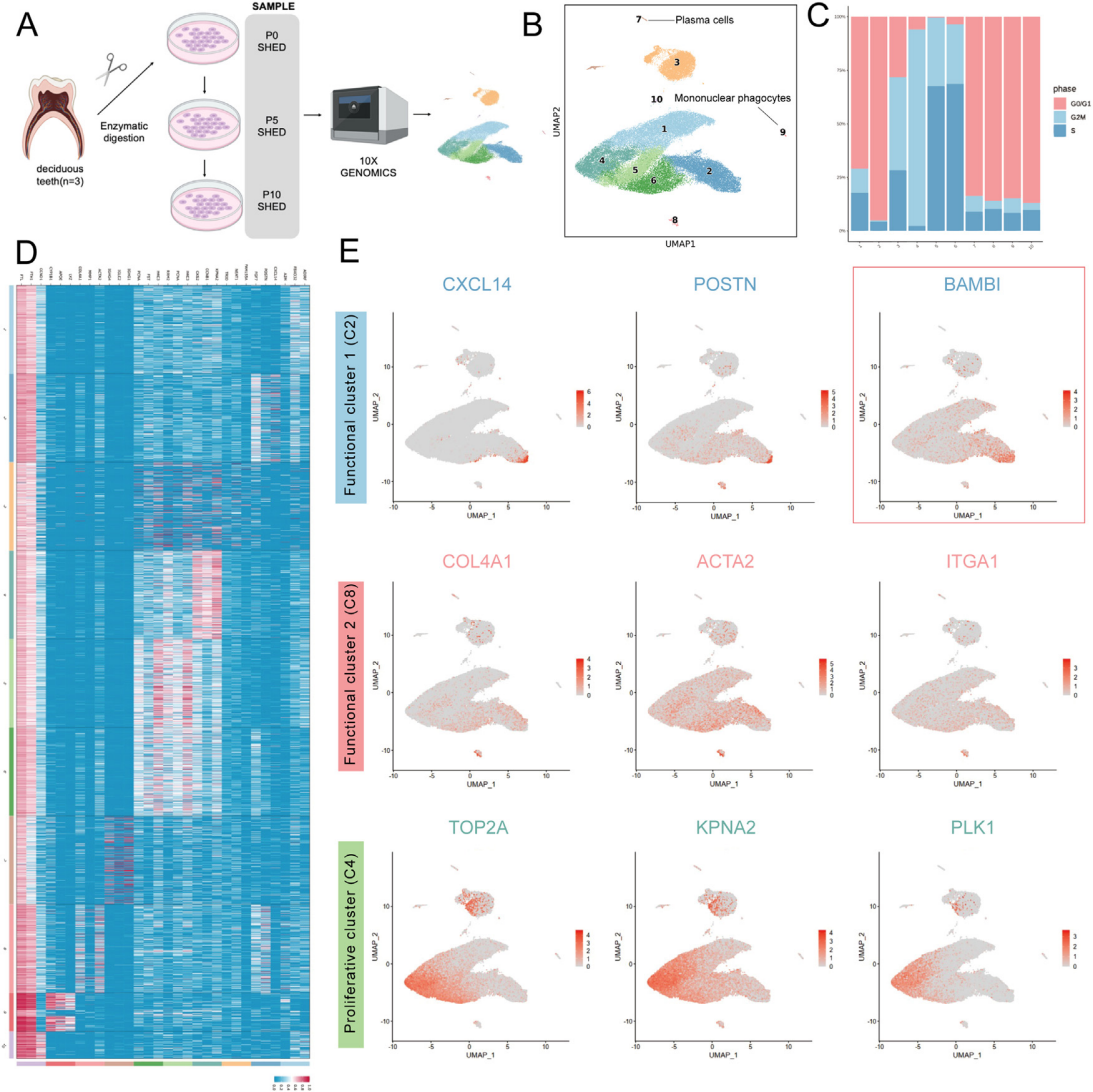
Single-cell RNA sequencing (scRNA-seq) of SHEDs allowed the identification of distinct subpopulations. (A) Workflow of the study. Pulp tissue was obtained from healthy human exfoliated deciduous teeth (n = 3). The tissues were cut into pieces followed by enzymatic digestion. P0 SHEDs were obtained after in vitro culture and expansion. After the cell suspensions were collected, a portion of the cells was passaged for further cultivation, successively yielding P5 and P10 SHEDs. Cells from these three passages (P0, P5 and P10) were subjected to scRNA-seq using the 10X Genomics platform. (B) UMAP visualization of 10 clusters of SHEDs from the three passages (n = 53286 cells) and SingleR annotation. (C) Percentages of SHED subpopulations in different cell cycle phases. (D) Heatmaps of the enriched genes identified from SHEDs. (E) Plots of the expression patterns of marker genes in the three critical clusters from the UMAP plots. Cells with low and high expression are indicated by grey and red, respectively.

To explore the composition of SHEDs, we first sequenced 53,286 cells from passage 0 (P0), passage 5 (P5), and passage 10 (P10) SHEDs via scRNA-seq (Figure 1 A). Stem cells isolated from the pulp of healthy human exfoliated deciduous teeth were analyzed via 10X Genomics scRNA-seq. After the sample was subjected to quality control, low-quality cells were removed to meet the needs of the subsequent bioconfidence analysis (Figure S1A).

The main cell populations were defined by the SingleR calculation method, ^13^ which involves the association of single-cell gene expression with the reference cell type marker datasets recorded in the human cell atlas. ^14^^,^^15^ As shown in Figure 1 B, unbiased clustering revealed 10 clusters, among which monocyte macrophages (cluster 9) and plasma cells (cluster 7) were separated from the other clusters. Excluding these 2 clusters, SHEDs highly expressed MSC positive markers such as CD90 (THY1), CD73 (NT5E), and CD105 (ENG) but lacked expression of MSC negative markers such as CD19, CD34, and CD45 (PTPRC) (Figure S1B). Considering that clusters 7 and 9 accounted for only 0.92% of the SHED population (Figure S1C), the cultured SHEDs were typical MSCs that met the ISCT standards. ^16^ Cluster 3 was highly associated with fibroblasts in the computational analysis; therefore, this subpopulation was defined as a fibroblast-like group. Cell cycle analysis revealed a significant difference in the cell cycle distribution ratio among different subpopulations; cluster 4 was in a state with a relatively high proportion of cells in the G2/M phase (Figure 1 C), thus defining it as a proliferative subpopulation. Hierarchical cluster analysis revealed close relationships among these 10 clusters (Figure 1 D). Owing to the presence of fewer marker genes or the absence of marker genes (differentially expressed genes with Log2FC < 1), clusters 1, 5 and 6 were defined as a transitional subgroup. Cluster 4 exhibited expression of genes related to proliferation and the cell cycle, further confirming our previous definition of cluster 4 as a proliferative subgroup. Clusters 2 and 8 presented expression of genes related to immune regulation, inflammatory processes, and tissue development and regeneration. Thus, they were identified as functional subpopulations 1 and 2. (Figure 1 E, Figure S2) Notably, the marker gene BAMBI that was expressed in functional subpopulation 1 (cluster 2) had a significantly higher expression level in cluster 2 than in the other subgroups. Among the filtered 25 genes of the corresponding DEG list (log2FC > 1), BAMBI showed the highest fold-change and, critically, encodes a cell-membrane-anchored protein, thereby enabling live sorting with a conventional antibody. The protein encoded by BAMBI is a membrane protein. Therefore, we selected BAMBI expression as the characteristic phenotype of cluster 2, which facilitated subsequent flow cytometric sorting of SHEDs for functional studies of different subpopulations. Cluster 10 contained very few cells, only 92 in total, and highly expressed genes FTH1 and FTL, related to iron metabolism (Figure 1 D, Figure S1C). It was inferred that these cells were a population of cells in a state of oxidative stress.

### GO and KEGG enrichment analyses of SHED subpopulations

**Fig. 2:**
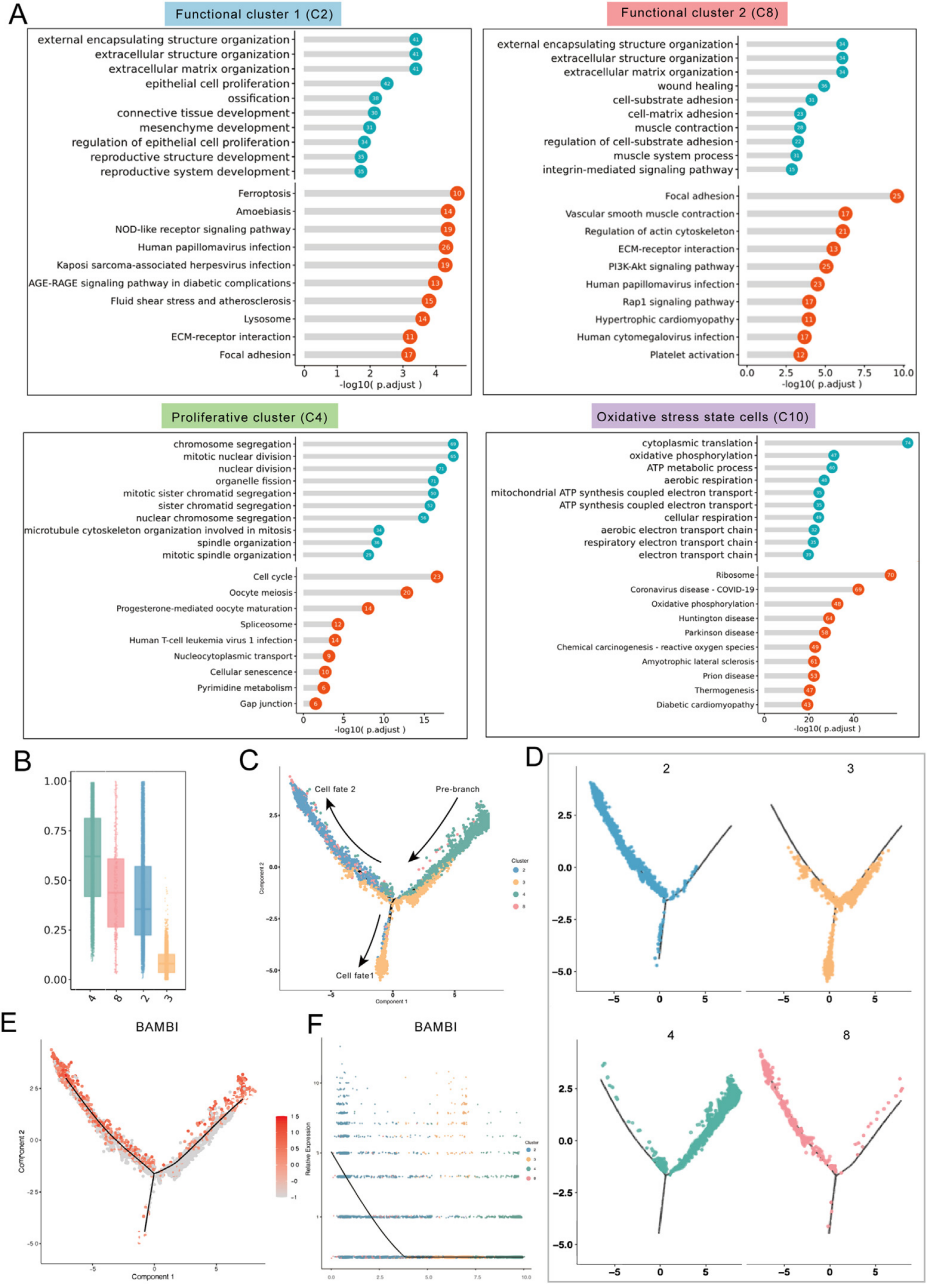
Enrichment analyses and transformation trajectory analysis of SHEDs subpopulations. (A) The enriched biological process terms determined by GO and KEGG analyses for different subpopulations. (B) Boxplot of the CytoTrace scores for different SHED subpopulations. The clusters are presented along the x-axis, and the CytoTrace scores are indicated on the y-axis. Higher CytoTrace scores indicate greater cell stemness and differentiation potential. (C, D) Monocle pseudotime trajectory branch showing the changes in progression of Clusters 2/3/4/8. (E, F) Pseudotime trajectory branch and expression dynamics along the pseudotime of BAMBI plotted by Monocle2.

To further analyse the possible functions of these SHED subpopulations, GO and KEGG enrichment analyses were conducted (Figure 2 A). In the biological process category, the marker genes of functional cluster 1 were enriched in epithelial cell proliferation, ossification, and connective tissue and mesenchyme development, indicating their multipotent differentiation ability. The marker genes of functional cluster 2 were enriched mainly in wound healing and several regulatory terms. In addition, in the biological process category, G2/M transition of the mitotic cell cycle was enriched in the proliferative cluster. The biological processes and signalling pathways of cluster 10 were related mainly to oxidative phosphorylation and reactive oxygen species, which is consistent with our previous speculation.

### Trajectory analysis of SHED subpopulations

Next, we selected clusters 2, 3, 4, and 8 to explore the differentiation trajectories of the SHED subpopulations. The CytoTRACE algorithm was used to score the differentiation levels, which revealed that the differentiation hierarchy from low to high was cluster 4 (proliferative cluster), cluster 8 (functional cluster 2), cluster 2 (functional cluster 1), and cluster 3 (fibroblast-like cells) (Figure 2 B). A trajectory with two branches corresponding to two different cell fates was constructed (Figure 2 C, D), with the proliferative cluster (C4) located at the root of the trajectory. Moreover, most fibroblast-like cells were positioned at the root of cell fate 1. The root of cell fate 2 was composed of functional clusters 1 and 2 (C2 and C8). Subsequently, pseudotime trajectory and gene expression dynamics analyses revealed that the phenotypic marker BAMBI of functional cluster 1 (C2) was upregulated in cell fate 2 (Figure 2 E, F). This finding also indicated that functional subpopulation 1 could be precisely selected as the BAMBI^+^ subpopulation. As previously described, BAMBI is predominantly expressed on the cell membrane; therefore, it was selected as a marker for functional subpopulation 1 in subsequent functional validation experiments.

### Biological performance of the BAMBI^+^ functional SHED subpopulation

**Fig. 3:**
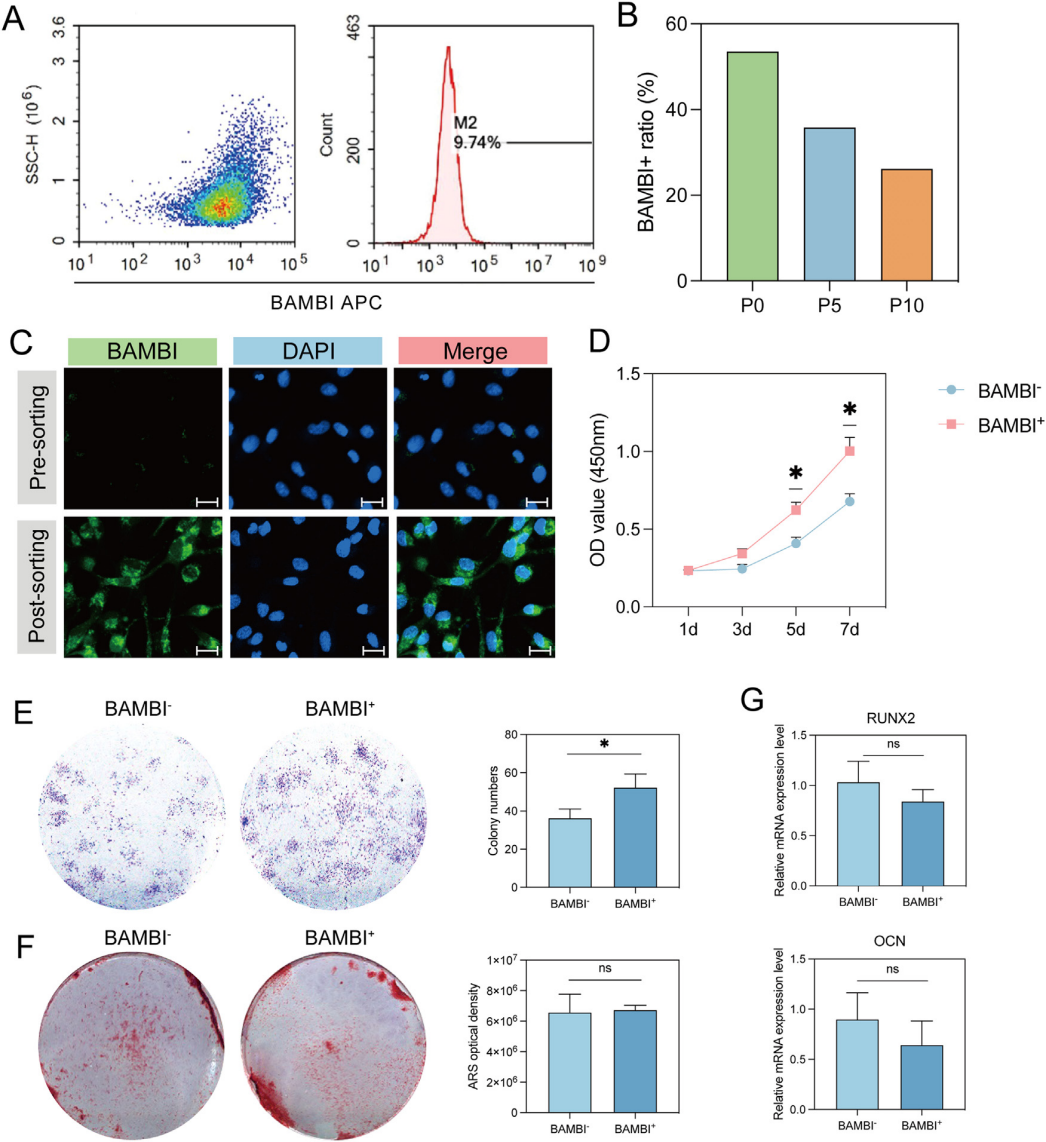
Biological performance of the BAMBI^+^ SHED functional subpopulation. (A) BAMBI^+^ SHEDs were sorted by flow cytometry. (B) Proportion of BAMBI^+^ cell in different generations of SHEDs. (C) BAMBI^+^ SHEDs before and after FACS. Scale bar: 20 μm. (D) Curves of the change in number of BAMBI^+^ and BAMBI^-^ SHEDs every 48 h over 7 days of expansion. (E) Colony formation assays with BAMBI^+^ and BAMBI^-^ SHEDs. (F) Alizarin Red S staining image and quantification of the mineralized matrix obtained from BAMBI^+^ and BAMBI^-^ SHEDs after 21 days of induction. (G) Expression of the osteogenic marker genes RUNX2 and OPN in BAMBI^+^ and BAMBI^-^ SHEDs after 7 days of culture. (^∗^*P* < 0.05, ns: *P* > 0.05)

Then, we sorted BAMBI^+^ SHEDs using flow cytometry to comprehensively study their biological properties (Figure 3 A). The results of scRNA-seq showed that with increasing generations, the proportion of BAMBI^+^ subsets decreased (Figure 3 B). Although each passage was represented by >10,000 cells, all three passages were processed in a single 10× run; therefore the passage comparison should be considered descriptive until independent biological replicates become available. Immunofluorescence staining revealed an increased proportion of BAMBI^+^ cells before and after fluorescence-activated cell sorting (FACS), confirming the successful isolation of the BAMBI^+^ subpopulation (Figure 3 C). We tracked the growth of the cells every 48 hours for 7 days. The results of the CCK-8 assay revealed that, compared with BAMBI^-^ cells, BAMBI^+^ cells presented greater proliferative capacity, which was confirmed by the continuous increase in cell number during the 7 days of in vitro culture (Figure 3 D). These results were confirmed by the colony formation assay (Figure 3 E).

**Fig. 4:**
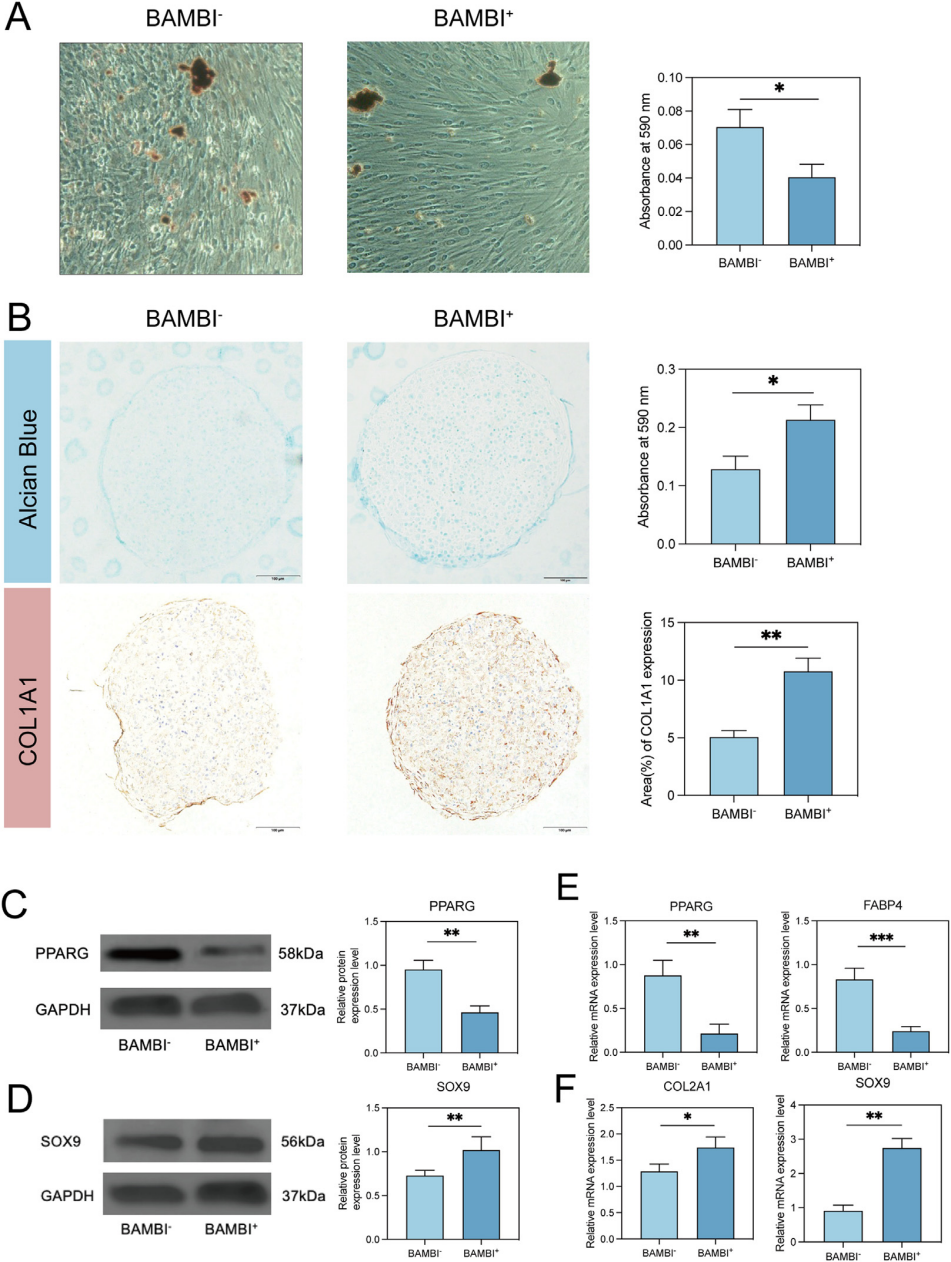
Biological performance of the BAMBI^+^ SHED functional subpopulation. (A) Oil red O staining image of the mineralized matrix obtained from BAMBI^+^ and BAMBI^-^ SHEDs after 28 days of induction. Scale bars: 200 μm. (B) Alcian blue staining image and immunostaining for collagen type I (COL1) of the cartilage pellets obtained from BAMBI^+^ and BAMBI^-^ SHEDs after 28 days of induction. Scale bars: 100 μm. (C) Western blot analysis of PPARG protein expression after 14 days of culture in adipogenic medium. (D) Western blot and quantitative analysis of SOX9 protein expression after 14 days of induction. (E) Expression of the adipogenic marker genes PPARG and FABP4 in BAMBI^+^ and BAMBI^-^ SHEDs after 7 days of culture. (F) Expression of the chondrogenic marker genes SOX9 and COL2A1 in BAMBI^+^ and BAMBI^-^ SHEDs after 7 days of culture. (^∗^*P* < 0.05, ^∗∗^*P* < 0.01, ^∗∗∗^*P* < 0.001).

We further investigated the osteogenic, adipogenic, and chondrogenic differentiation potential of the BAMBI^+^ subpopulation. After 1–3 weeks of osteogenic induction, no significant differences were observed between BAMBI^+^ and BAMBI^-^ SHEDs, which was confirmed by the lack of significant changes in osteogenic lineage markers at the mRNA levels (Figure 3 G). The results of Alizarin Red S (ARS) staining and quantitative analysis were consistent (Figure 3 F). In addition, compared with BAMBI^-^ cells, BAMBI^+^ SHEDs presented weaker adipogenic differentiation potential, as evidenced by the significantly lower expression of the adipogenic marker PPARG at both the mRNA and protein levels (Figure 4 C, E). After 7 days of induction, the expression of the adipogenic-related gene FABP4 was also downregulated, which was consistent with the detection of fewer lipid droplets by Oil Red O staining (Figure 4 A). In contrast, the BAMBI^+^ subpopulation presented increased chondrogenic differentiation potential, as confirmed by high expression of chondrogenic markers such as SOX9 and COL2A1 (Figure 4 D, F). Moreover, the immunohistochemical results, as well as increased glycosaminoglycan content, revealed that the BAMBI^+^ subpopulation presented increased expression of COL1, which was consistent with previous findings (Figure 4 B).

### Proangiogenic potential of the BAMBI^+^ SHED functional subpopulation

**Fig. 5:**
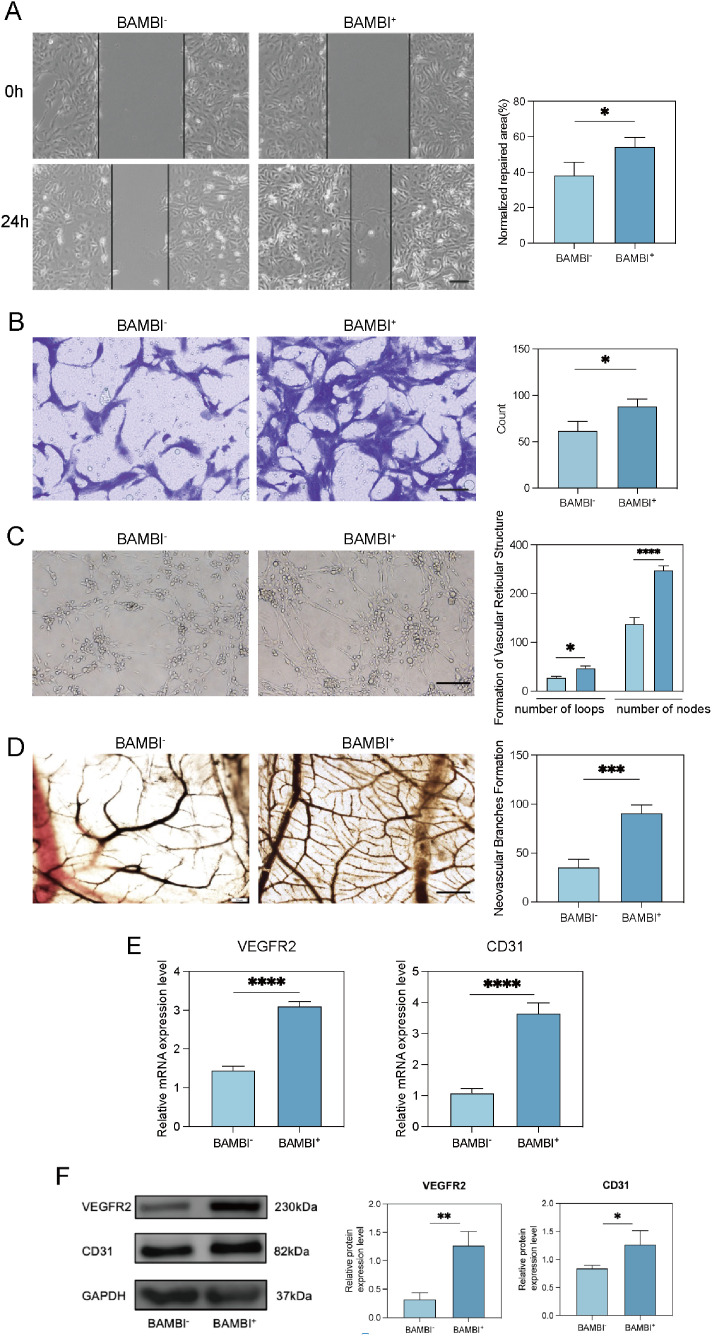
Proangiogenic potential of the BAMBI^+^ SHED functional subpopulation. (A) Repair of the scratch area of HUVECs cultured in BAMBI^+^ or BAMBI^-^ SHED cell culture media and quantitative analysis. Scale bars: 500 μm. (B) HUVEC migration influenced by BAMBI^+^ and BAMBI^-^ SHEDs and quantitative analysis. Scale bars: 100 μm. (C) In vitro tubule formation by BAMBI^+^ and BAMBI^-^ SHEDs and quantitative analysis of the loops and nodes. Scale bars: 200 μm. (D) CAM assay to evaluate neovascular formation and quantitative analysis. Samples were stimulated with BAMBI^+^ or BAMBI^-^ SHED cell culture media. Scale bars: 200 μm. (E) Expression of VEGFR2 and CD31 in BAMBI^+^ and BAMBI^-^ SHEDs after 7 days of induction. (F) Western blot and quantitative analysis of VEGFR2 and CD31 protein expression. (^∗^*P* < 0.05, ^∗∗^*P* < 0.01, ^∗∗∗^*P* < 0.001, ^∗∗∗∗^*P* < 0.0001).

To determine whether the BAMBI^+^ subpopulation has unique functional characteristics, we also tested its angiogenic capacity. Previous scRNA-seq data revealed that cluster 2 exhibited expression of genes related to angiogenesis and connective tissue development (Figure 2 A). After 7 days of induction, the qPCR results indicated that, compared with BAMBI^-^ cells, BAMBI^+^ SHEDs presented greater expression of genes associated with angiogenesis (VEGFR2 and CD31) (Figure 5 E). Western blot analysis revealed that compared with BAMBI- cells, BAMBI+ SHEDs exhibited higher expression of the angiogenesis-related proteins VEGFR2 and CD31 (Figure 5F). Scratch tests and Transwell assays (Figure 5 A, B) demonstrated the effects of culture medium from BAMBI^+^ or BAMBI^-^ SHEDs on the migration ability of HUVECs. Compared with that in the BAMBI^-^ group, the migration distance of HUVECs in the BAMBI^+^ group was significantly greater. Similarly, Matrigel tube formation assays revealed that BAMBI^+^ cells formed more reticular loops and nodes (Figure 5 C), indicating their greater in vitro angiogenic capacity. In addition, CAM assays revealed that BAMBI^+^ SHEDs formed a greater number of blood vessels, demonstrating their enhanced in vivo angiogenic ability (Figure 5 D).

### Profiles of SHEDs at different passages by scRNA-seq analysis

**Fig. 6:**
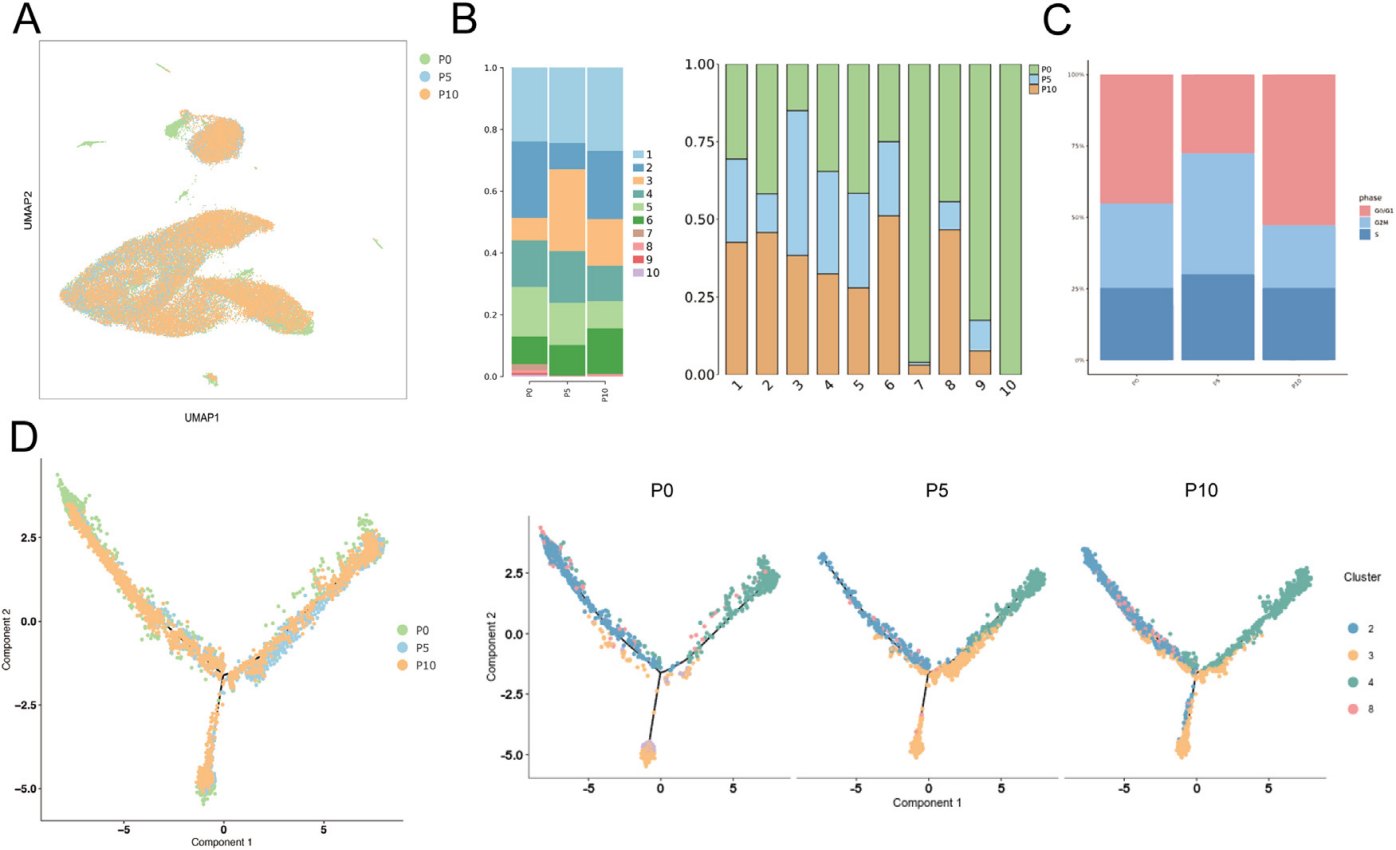
Profiles of SHEDs from different passages on the basis of single-cell sequencing. (A) UMAP visualization of SHEDs at different passages. (B) Percentages of SHEDs at different passages in the ten clusters and different clusters of the three passages. (C) Percentages of SHEDs at different passages in different phases of the cell cycle. (D) Monocle pseudotime trajectory branch of SHEDs at different passages.

To evaluate the differences among different generations of SHEDs, scRNA-seq data were used to analyse P0, P5, and P10, with dimensionality reduction clustering shown in Figure 6 A. We found that functional subpopulation 1 (cluster 2) appeared stable in all three generations, and its proportion was highest in the P0 generation. Clusters 7, 9, and 10 were present mainly in the P0 generation, and cluster 10 was found only in P0. These findings indicate that these immune cells and cells in a state of oxidative stress cannot be maintained at stable proportions through passage in culture (Figure 6 B). Cell cycle analysis revealed that, compared with those in P0 and P10, the proportion of cells in the G2/M phase was highest in the P5 generation, with the most active cell division and proliferation (Figure 6 C). Pseudotime analysis revealed that SHEDs from different generations had similar cell fate trajectories. Among them, the proportion of P0 generation cells heading towards cell fate root 1 dominated by fibroblast-like cells was small, whereas the P10 generation had a larger proportion (Figure 6 D).

## Discussion

In this study, we used scRNA-seq technology to identify SHED clusters, including a BAMBI^+^ functional subpopulation, a proliferative subpopulation, fibroblast-like cells, and cells in a state of oxidative stress. Through trajectory analysis, we determined that the proliferative subpopulation differentiated into either a functional subpopulation or fibroblast-like cells. Further research revealed that the BAMBI^+^ functional subpopulation was more capable of promoting angiogenesis, chondrogenic differentiation, and proliferation than the other subpopulations but had a lower potential for adipogenic differentiation. By comparing the scRNA-seq data of SHEDs at different passages, we found that the proportion of the functional subpopulation (cluster 2) was highest in the P0 generation. In addition, SHEDs from different generations had similar cell fate trajectories.

As a unique population of craniofacial neural crest-derived MSCs, SHEDs have become an important cell source for regenerative medicine because of their ease of acquisition, high proliferation capacity, and multipotent differentiation potential. However, for a long time, research on SHEDs has been based primarily on population-based cell analyses, and their cellular heterogeneity and mechanisms of functional specialization have not been elucidated. Compared with other types of MSCs (such as bone marrow MSCs or umbilical cord MSCs), single-cell studies of SHEDs have been severely lacking. 17, 18, 19, 20 Recently, large-scale single-cell databases have gradually included data from oral-derived stem cells, including dental pulp stem cells from permanent teeth (DPSCs), periodontal ligament stem cells (PDLSCs), and stem cells from the apical papilla (SCAPs), ^10^^,^^15^^,^^21^, ^22^, ^23^, ^24^ but data on deciduous tooth stem cells are scarce. ^25^ In this study, we constructed a high-resolution single-cell transcriptional atlas of SHEDs, delineated the transformation trajectories of various subgroups, and filled a critical gap in the field. This study not only advances the development of oral stem cell biology but also provides a methodological paradigm for the use of single-cell technologies to dissect other stem cell populations.

The core marker genes identified in this study reflect both conservation and lineage specificity compared with other MSC studies at multiple levels. First, SHEDs highly express the MSC positive markers CD90, CD73, and CD105 but lack the expression of MSC negative markers, such as CD19, CD34, and CD45 (PTPRC). Additionally, the BAMBI^+^ subpopulation in SHED cluster 2 also expresses other characteristic markers of DPSCs, such as SOX4, which is consistent with the study by Yin et al (Figure S1D). ^11^ As a functional subpopulation, it also expresses markers of the MSC functional subpopulation, such as IGFBP2; the proliferative subpopulation (cluster 4) expresses the proliferative subpopulation marker of MSCs, PLK1 (Figure S1D). ^7^ Overall, our data are consistent with those of previous studies in which MSCs from different individuals were used, which demonstrates the reproducibility of our sequencing data.

In this study, a fibroblast-like subpopulation (cluster 3) with high expression of collagen synthesis genes was identified through single-cell transcriptomic analysis. Trajectory analysis suggested that this subpopulation serves as a cell fate root and is in a more differentiated state. Fibroblasts exhibit morphology, surface marker expression, proliferation, differentiation, and immune regulatory capabilities similar to those of MSCs. 26, 27, 28, 29 These similarities not only blur cellular identities but also limit the applications of the cells. Soundararajan et al. reported that fibroblast surface markers are expressed in ageing MSCs. ^30^ Other studies have shown that fibroblasts do not represent ageing MSCs and can differentiate from MSCs under specific conditions. ^31^^,^^32^ Despite these conflicting findings, it is generally accepted that fibroblasts are closely related to MSCs. Scholars have used scRNA-seq technology to analyse the correlation between MSCs and fibroblasts and have reported that cell subpopulations with an MSC phenotype also exhibit a fibroblast phenotype. However, subpopulations with a fibroblast phenotype do not necessarily exhibit an MSC phenotype, indicating that MSCs represent a subclass of fibroblasts. ^33^ Our study supports the hypothesis that MSCs and fibroblasts share a core molecular program, providing new evidence for the theory that MSCs are actually specialized fibroblast-like cells. However, it also highlights the limitations of currently used cell definition criteria.

The functional subpopulation cluster 2, which expresses various cytokines at high levels, attracted our attention. Among the evaluated marker genes, BAMBI was selected as a marker for this functional subpopulation. This study is the first to reveal the central role of the BAMBI^+^ subpopulation of SHEDs at the single-cell level. BAMBI (BMP and activin membrane-bound inhibitor) encodes a transmembrane glycoprotein related to type I receptors of the transforming growth factor-β (TGF-β) family, which play important roles in signal transduction during many developmental and pathological processes. Adipogenesis experiments have shown that this subpopulation has significantly reduced potential for adipogenic differentiation. This finding is consistent with another scRNA-seq study of MSCs and the inhibition of adipogenesis in BAMBI-expressing MSCs. 34, 35 Moreover, for the first time, we found that the BAMBI^+^ subpopulation has stronger chondrogenic differentiation and angiogenic promotion capabilities than the other subpopulations. In terms of chondrogenesis, this may be due to the prevention of pathological calcification by inhibiting excessive activation of BMP on the one hand and the promotion of SOX9 expression and type II collagen deposition by activating the noncanonical TGF-β pathway on the other hand. 36, 37 In terms of angiogenesis, BAMBI can modulate alternative pathways of the TGF-β signalling pathway, thereby affecting the homeostasis of vascular endothelial cells and angiogenesis. Additionally, BAMBI can increase the transcriptional activity of the Wnt signalling pathway, thereby promoting cell proliferation and angiogenesis. 38, 39 Interestingly, research has indicated a negative correlation between the immunomodulatory and proangiogenic capacities of MSCs and that this correlation can be used to predict interdonor differences in proangiogenic and anti-inflammatory/immunosuppressive activities in cell-based experiments. ^40^ In conjunction with the findings of Chen et al., the BAMBI^high^ MFGE8^high^ MSC subpopulation exhibited decreased immunosuppressive activity in vitro and in vivo in lupus mice. ^34^ These data are in line with our experimental results showing that the BAMBI^+^ subpopulation exhibits proangiogenic capabilities. Our findings demonstrate that the BAMBI⁺ SHED subpopulation exhibits strong pro-angiogenic capacity, as evidenced by enhanced tube formation and HUVEC migration. Interestingly, a recent study by Zhu et al. also highlighted the importance of direct cell contact and ATP1A1-mediated Src/AKT signaling in stabilizing vascular networks formed by DPSCs and endothelial cells, suggesting that similar transmembrane proteins may orchestrate intercellular crosstalk in dental stem cell-driven angiogenesis ^41^. Future studies could explore whether BAMBI interacts with such pathways to fine-tune vascularization in SHED-based regeneration.

While we observed that the BAMBI⁺ subpopulation did not exhibit enhanced osteogenic differentiation compared to BAMBI⁻ cells, it is noteworthy that extrinsic factors such as magnesium ions have been shown to potently enhance the osteogenic capacity of other dental stem cells like PDLSCs via TRPM7 channels ^42^. This suggests that the differentiation potential of SHEDs may be further modulated by specific ionic microenvironments, which could be leveraged in future scaffold-based regenerative strategies.

Clinical treatment requires many MSCs, necessitating in vitro cell expansion. Although MSCs possess self-renewal and multipotent differentiation abilities, these abilities tend to decline over time when the cells are cultured in vitro. With the onset of cell cycle arrest and the loss of proliferative capacity, some MSCs may undergo senescence. ^43^ However, there is a debate regarding whether the MSCs at the first passage result in the strongest effects. For example, Xie et al. reported that the proportions of functional subpopulations were greater in third-passage MSCs than in first-passage MSCs. ^7^ To investigate this issue, we compared the scRNA-seq data of SHEDs across three different passages. The results indicated that the proportion of the functional subpopulation was highest in the P0 generation and that the proportion of the BAMBI^+^ subpopulation decreased with increasing generations. However, the proportion of cluster 2 did not decrease with increasing passage number. Therefore, we similarly suggest that the function of SHEDs primarily depends on the proportions of functional subpopulations at the time of assessment rather than the number of passages. Moreover, directly isolating the BAMBI^+^ functional subpopulation or promoting the differentiation of stem cell subpopulations into functional subpopulations may hold promise for clinical application.

## Conclusion

In summary, in this study, we constructed a new comprehensive atlas of SHEDs using single-cell transcriptomics analysis and delineated the transformation trajectories of the cells. More importantly, we discovered the unique functions of the BAMBI^+^ subpopulation. This study will aid in the selection of the optimal type of MSCs for treating specific diseases and increase their clinical efficacy. Future research needs to further integrate spatial transcriptomics analysis to elucidate the in situ behaviour of SHED subpopulations in tissue regeneration and conduct large-animal model studies to assess the extent to which sorting of subpopulations improves regenerative outcomes. Additionally, exploring whether there are similar BAMBI^+^ functionally equivalent subpopulations in MSCs from different tissue sources will support the establishment of a unified theoretical framework for cross-organ regenerative medicine.

## Ethical approval

The study was conducted according to the guidelines of the Declaration of Helsinki. All experimental protocols were approved by the Ethics Committee of the Peking University Health Science Center, Beijing, China (ethical approval number: PKUSSIRB-202394170).

## Declaration of competing interest

The authors declare that they have no known competing financial interests or personal relationships that could have appeared to influence the work reported in this paper.

## Appendix Supplementary materials

Image, application 1

## References

[1] Miura M., Gronthos S., Zhao M., Lu B., Fisher L.W., Robey P.G. (2003). SHED: Stem cells from human exfoliated deciduous teeth. Proc Natl Acad Sci USA.

[2] Xuan K., Li B., Guo H. (2018). Deciduous autologous tooth stem cells regenerate dental pulp after implantation into injured teeth. Sci Transl Med.

[3] Nakamura S., Yamada Y., Katagiri W., Sugito T., Ito K., Ueda M. (2009). Stem cell proliferation pathways comparison between human exfoliated deciduous teeth and dental pulp stem cells by gene expression profile from promising dental pulp. J Endod.

[4] Ko C.S., Chen J.H., Su WT. (2020). Stem Cells from Human Exfoliated Deciduous Teeth: A Concise Review. Curr Stem Cell Res Ther.

[5] Kim J.H., Kim G.H., Kim J.W., Pyeon HJ2, Lee J.C., Lee G. (2016). In vivo angiogenic capacity of stem cells from human exfoliated deciduous teeth with human umbilical vein endothelial cells. Mol Cells.

[6] Stuart T., Satija R. (2019). Integrative single-cell analysis. Nat. Rev. Genet..

[7] Xie Z., Yu W., Ye G. (2022). Single-cell RNA sequencing analysis of human bone-marrow-derived mesenchymal stem cells and functional subpopulation identification. Exp Mol Med.

[8] Zhang S., Wang J.Y., Li B., Yin F., Liu H. (2021). Single-cell transcriptome analysis of uncultured human umbilical cord mesenchymal stem cells. Stem Cell Res Ther.

[9] Liu Q., Zhao Y., Wang Q., Yan L., Fu X., Xiao R. (2023). Convergent alteration of the mesenchymal stem cell heterogeneity in adipose tissue during aging. FASEB J.

[10] Cui Y., Ji W., Gao Y., Xiao Y., Liu H., Chen Z. (2021). Single-cell characterization of monolayer cultured human dental pulp stem cells with enhanced differentiation capacity. Int J Oral Sci.

[11] Yin W., Liu G., Li J., Bian Z. (2021). Landscape of cell communication in human dental pulp. Small Methods.

[12] Wang D., Zhu N., Xie F., Qin M., Wang Y. (2022). Long non-coding RNA IGFBP7-AS1 accelerates the odontogenic differentiation of stem cells from human exfoliated deciduous teeth by regulating IGFBP7 expression. Hum Cell.

[13] Aran D., Looney A.P., Liu L. (2019). Reference-based analysis of lung single-cell sequencing reveals a transitional profibrotic macrophage. Nat Immunol.

[14] Krivanek J., Soldatov R.A., Kastriti M.E. (2020). Dental cell type atlas reveals stem and differentiated cell types in mouse and human teeth. Nat Commun.

[15] Pagella P., de Vargas Roditi L., Stadlinger B., Moor A.E., Mitsiadis TA. (2021). A single-cell atlas of human teeth. iScience.

[16] Dominici M., Le Blanc K., Mueller I. (2006). Minimal criteria for defining multipotent mesenchymal stromal cells. The International Society for Cellular Therapy position statement. Cytotherapy.

[17] Wang Q., Li J., Wang S. (2021). Single-cell transcriptome profiling reveals molecular heterogeneity in human umbilical cord tissue and culture-expanded mesenchymal stem cells. FEBS J.

[18] Wang Z., Li X., Yang J. (2021). Single-cell RNA sequencing deconvolutes the in vivo heterogeneity of human bone marrow-derived mesenchymal stem cells. Int J Biol Sci.

[19] Wang Z., Chai C., Wang R. (2021). Single-cell transcriptome atlas of human mesenchymal stem cells exploring cellular heterogeneity. Clin Transl Med.

[20] Zhang S., Wang J.Y., Li B., Yin F., Liu H. (2021). Single-cell transcriptome analysis of uncultured human umbilical cord mesenchymal stem cells. Stem Cell Res Ther.

[21] Chen D., Park M. (2022). Single-cell RNA sequencing analysis of human dental pulp stem cell and human periodontal ligament stem cell. J Endod.

[22] Weng Y., Xiao Y., Shi Y. (2025). A single-cell transcriptomic atlas of human stem cells from apical papilla during the committed differentiation. Int Endod J.

[23] Qian Y., Gong J., Lu K. (2023). DLP printed hDPSC-loaded GelMA microsphere regenerates dental pulp and repairs spinal cord. Biomaterials.

[24] Pinkhasov I., Kabakov L., Nemcovsky C.E. (2023). Single-cell transcriptomic analysis of oral masticatory and lining mucosa-derived mesenchymal stromal cells. J Clin Periodontol.

[25] Li Y., Song G., Jiang Y. (2024). Single-cell transcriptome analysis of stem cells from human exfoliated deciduous teeth investigating functional heterogeneity in immunomodulation. Sci Rep.

[26] Alt E., Yan Y., Gehmert S., Song Y.-H., Altman A., Gehmert S. (2011). Fibroblasts Share Mesenchymal Phenotypes with Stem Cells, but Lack Their Differentiation and colony-forming Potential. Biol. Cel.

[27] Zhang W.J., Bi D., Liu W., Wei X., Chen F.F. (2007). Clonal Analysis of Nestin(-) Vimentin(+) Multipotent Fibroblasts Isolated from Human Dermis. J. Cel Sci..

[28] Haniffa M.A., Wang X.-N., Holtick U., Rae M., Isaacs J.D., Dickinson A.M. (2007). Adult human fibroblasts are potent immunoregulatory cells and functionally equivalent to mesenchymal stem cells. J. Immunol..

[29] Blasi A., Martino C., Balducci L., Saldarelli M., Soleti A., Navone S.E. (2011). Dermal Fibroblasts Display Similar Phenotypic and Differentiation Capacity to Fat-Derived Mesenchymal Stem Cells, but Differ in Antiinflammatory and Angiogenic Potential. Vasc. Cel.

[30] Soundararajan M., Kannan S. (2018). Fibroblasts and mesenchymal stem cells: two sides of the same coin. J. Cel. Physiol..

[31] Mishra P.J., Mishra P.J., Glod J.W., Banerjee D. (2009). Mesenchymal Stem Cells: Flip Side of the coin. Cancer Res..

[32] Chan T.-S., Shaked Y., Tsai K.K. (2019). Targeting the interplay between cancer fibroblasts, mesenchymal stem cells, and cancer stem cells in desmoplastic cancers. Front. Oncol..

[33] Fan C., Liao M., Xie L. (2022). Single-cell transcriptome integration analysis reveals the correlation between mesenchymal stromal cells and fibroblasts. Front Genet.

[34] Chen H., Wen X., Liu S. (2022). Dissecting Heterogeneity Reveals a Unique BAMBIhigh MFGE8high Subpopulation of Human UC-MSCs. Adv Sci (Weinh).

[35] Chen X., Zhao C., Xu Y. (2021). Adipose-specific BMP and activin membrane-bound inhibitor (BAMBI) deletion promotes adipogenesis by accelerating ROS production. J Biol Chem.

[36] Fongsodsri K., Tiyasatkulkovit W., Chaisri U. (2024). Sericin promotes chondrogenic proliferation and differentiation via glycolysis and Smad2/3 TGF-β signaling inductions and alleviates inflammation in three-dimensional models. Sci Rep.

[37] Weißenberger M., Weißenberger M.H., Wagenbrenner M. (2020). Different types of cartilage neotissue fabricated from collagen hydrogels and mesenchymal stromal cells via SOX9, TGFB1 or BMP2 gene transfer. PLoS One.

[38] Guillot N., Kollins D., Gilbert V. (2012). BAMBI regulates angiogenesis and endothelial homeostasis through modulation of alternative TGFβ signaling. PLoS One.

[39] Lin Z., Gao C., Ning Y., He X., Wu W., Chen YG. (2008). The pseudoreceptor BMP and activin membrane-bound inhibitor positively modulates Wnt/beta-catenin signaling. J Biol Chem.

[40] Robb K.P., Audet J., Gandhi R., Viswanathan S. (2022). Putative critical quality attribute matrix identifies mesenchymal stromal cells with potent immunomodulatory and angiogenic "fitness" ranges in response to culture process parameters. Front Immunol.

[41] Zhu M., Jiang S., Zhang C., Wu B., Zou T. (2025). ATP1A1-driven intercellular contact between dental pulp stem cell and endothelial cell enhances vasculogenic activity. Int Dent J.

[42] Lumbikananda S., Tikkhanarak K., Pongjantarasatian S., Trachoo V., Namangkalakul W., Osathanon T. (2025). Osteogenic induction activity of magnesium chloride on human periodontal ligament stem cells. Int Dent J.

[43] Turinetto V., Vitale E., Giachino C. (2016). Senescence in human mesenchymal stem cells: functional changes and implications in stem cell-based therapy. Int J Mol Sci.

