# Supplementary material for: Dissecting Heterogeneity Reveals Functional Subpopulation of Stem Cells From Human Exfoliated Deciduous Teeth
Source: Int Dent J. 2025 Nov 6;76(1):104014. doi: 10.1016/j.identj.2025.104014 (PMC12828145; doi:10.1016/j.identj.2025.104014)
Supplement: Supplementary file 1 [file mmc1.docx]

**Supplementary materials**

**Supplementary Table. List of primers’ sequences used in this article.**

| Primer | Sequence (5’-3’) |
| --- | --- |
| *GAPDH* | Forward: TGCACCACCAACTGCTTAGC |
|  | Reverse: GGCATGGACTGTGGTCATGAG |
| *RUNX2* | Forward: CACGGAGCACAGGAAGTTGGG |
|  | Reverse: AAGATGGATTGCACGCAGGTTCTC |
| *OCN* | Forward: TCACACTCCTCGCCCTATTG |
|  | Reverse: CTCTTCACTACCTCGCTGCC |
| *VEGFR2* | Forward: ACGGACAGTGGTATGGTTCTTGCC |
|  | Reverse: GGTAGCCGCTTGTCTGGTTTGAG |
| *CD31* | Forward: TGTCAAGTAAGGTGGTGGAGTCT |
|  | Reverse: AGGCGTGGTTGGCTCTGTT |
| *SOX9* | Forward: GACAGCCCCCTATCGACTTC |
|  | Reverse: CAAACTCGTTGACATCGAAGG |
| *COL2A1* | Forward: CAAGAACAGCATTGCCTATCTG |
|  | Reverse: GATAACAGTCTTGCCCCACTTA |
| *PPARG* | Forward: TGCTGGAGCCACAAAC |
|  | Reverse: AAACCCTATGCAACCTTC |
| *FABP4* | Forward: GGCCAAACCTAACATGATCATC |
|  | Reverse: TTATGGTGCTCTTGACTTTCCT |


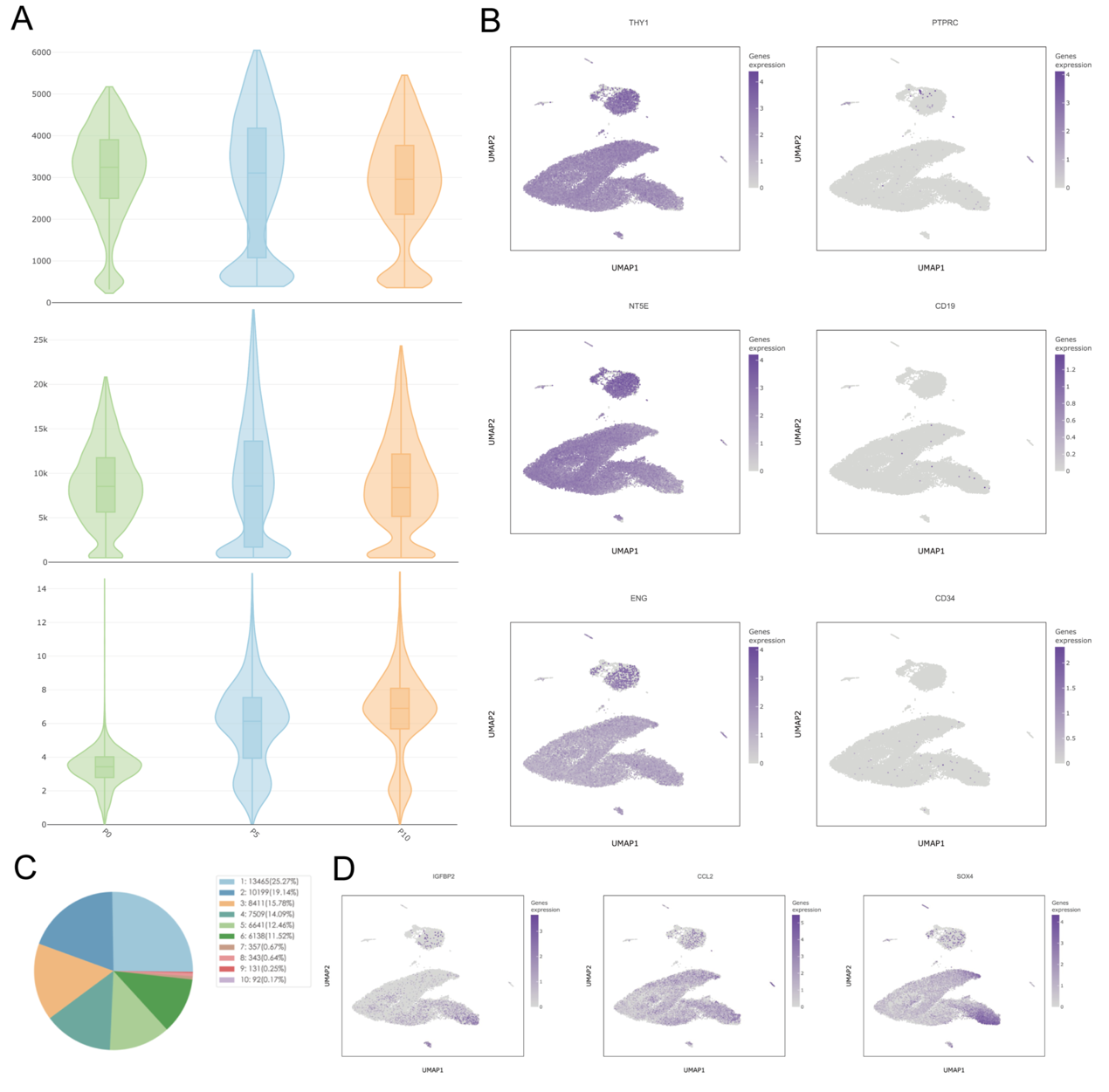


**Fig. S1 - (A) Violin plot showing the distribution of number of genes per cell, number of UMIs per cell, and proportion of mitochondrial genes in each cell of the indicated sample type after QC filtering. (B) Expression patterns of MSC-positive markers (THY1, NT5E, and ENG) and MSC-negative markers (CD19, CD34, and PTPRC) in SHEDs determined by scRNA-seq. (C) Pie chart showing the numbers and proportions of cells that constitute the different subpopulations. (D) Expression patterns of IGFBP2, CCL2 and SOX4.**

**
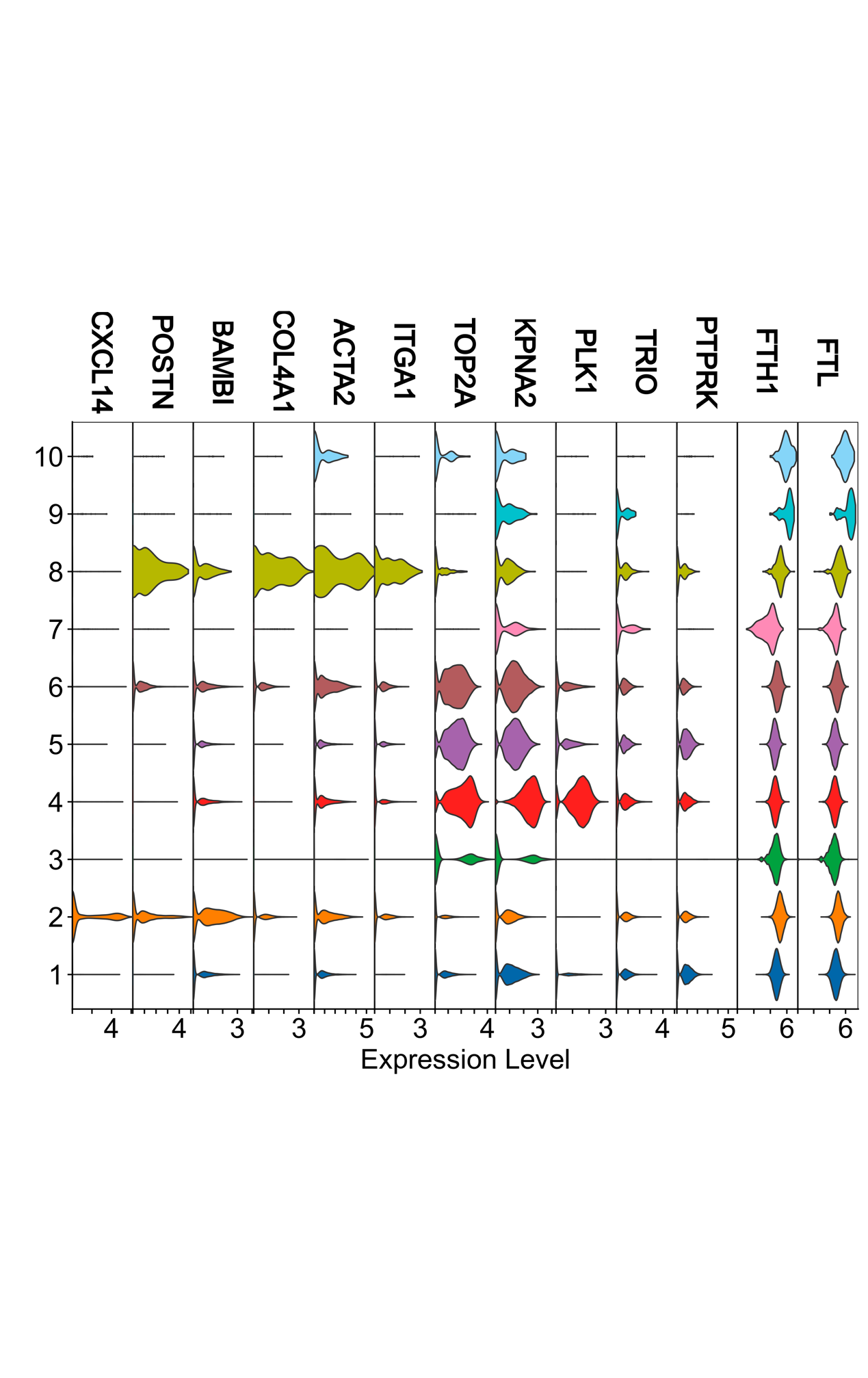
**

**Fig. S1 -** **Violin plots of key subpopulation marker genes.**
